# Supplementary material for: Measuring PETase enzyme kinetics by single-molecule microscopy
Source: bioRxiv. 2024 Apr 27:2024.04.24.590935. Preprint. [Version 2] doi: 10.1101/2024.04.24.590935 (PMC11071475; doi:10.1101/2024.04.24.590935)
Supplement: Supplement 1 [file NIHPP2024.04.24.590935v2-supplement-1.pdf]

bioRxiv preprint doi: https://doi.org/10.1101/2024.04.24.590935; this version posted April 27, 2024. The copyright holder for this preprint (which was not certified by peer review) is the author/funder, who has granted bioRxiv a license to display the preprint in perpetuity. It is made available under aCC-BY 4.0 International license.

Supporting Information

Measuring PETase enzyme kinetics by single-molecule microscopy

Yuwei Zhang and William O. Hancock

Departments of Chemistry and Biomedical Engineering, Pennsylvania State University, University Park, Pennsylvania, USA

Figures S1-S2.

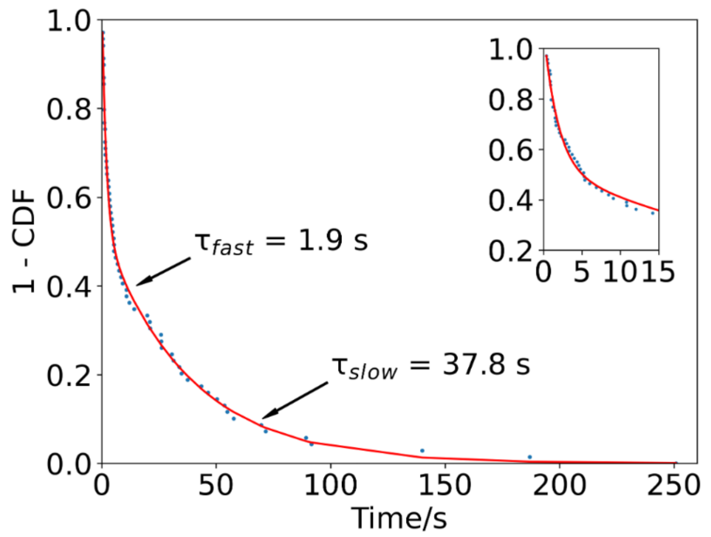

Figure S1 (related to Fig. 3): Binding durations of PETase-free Qdot control. Distribution of binding durations of Qdot control, fit by a biexponential. Inset: first 15 s to show details of fast phase.

bioRxiv preprint doi: https://doi.org/10.1101/2024.04.24.590935; this version posted April 27, 2024. The copyright holder for this preprint (which was not certified by peer review) is the author/funder, who has granted bioRxiv a license to display the preprint in perpetuity. It is made available under aCC-BY 4.0 International license.

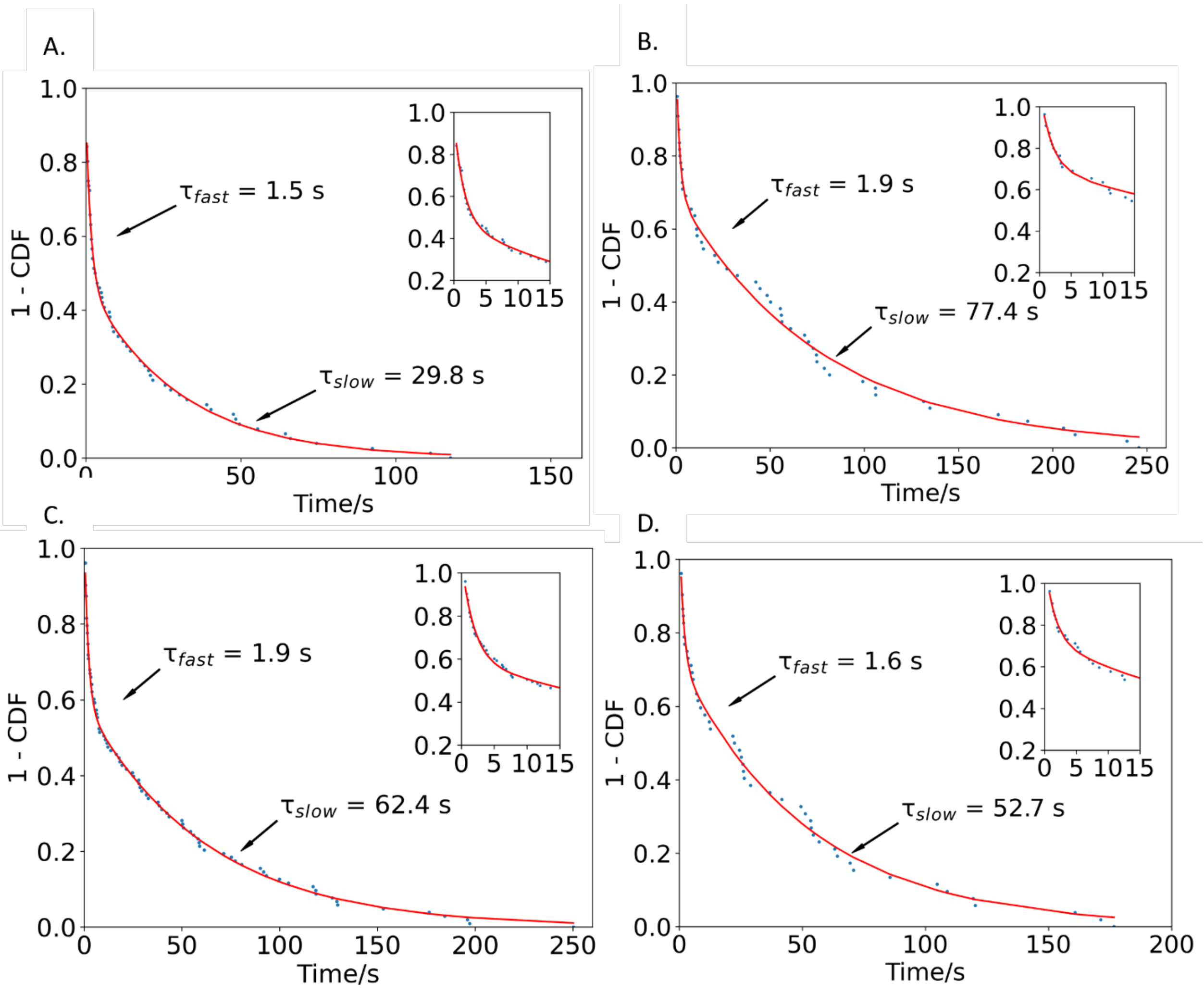

Figure S2 (related to Fig. 4): **Binding durations in the presence of 4 mM pNPA.** (A) Distribution of binding durations of Qdot control in the presence of 4 mM pNPA, fit by a biexponential. Inset: first 15 s to show details of fast phase. (B) Distribution of binding durations of wild-type PETase in the presence of 4 mM pNPA, fit by a biexponential. Inset: first 15 s to show details of fast phase. (C) Distribution of binding durations of the inactive PETase mutant in the presence of 4 mM pNPA, fit by a biexponential. Inset: first 15 s to show details of fast phase. (D) Distribution of binding durations of the hyperactive PETase mutant in the presence of 4 mM pNPA, fit by a biexponential. Inset: first 15 s to show details of fast phase.
